# Therapeutic Potential of Targeting p27^kip1^ in Plaque Vulnerability

**DOI:** 10.26502/aimr.0167

**Published:** 2024-04-22

**Authors:** Jerry Trinh, Jennifer Shin, Vikrant Rai, Devendra K. Agrawal

**Affiliations:** 1 Department of Translational Research, Western University of Health Sciences, Pomona CA 91766, USA

**Keywords:** Atherosclerosis, mTOR, p27^kip^, Oncostatin M, Plaque vulnerability, VSMC proliferation

## Abstract

Atherosclerosis, a critical contributor to coronary artery diseases, involves the accumulation of cholesterol, fibrin, and lipids within arterial walls, inciting inflammatory reactions culminating in plaque formation. This multifaceted interplay encompasses excessive fibrosis, fatty plaque development, vascular smooth muscle cell (VSMC) proliferation, and leukocyte migration in response to inflammatory pathways. While stable plaques demonstrate resilience against complications, vulnerable ones, with lipid-rich cores, necrosis, and thin fibrous caps, lead to thrombosis, myocardial infarction, stroke, and acute cerebrovascular accidents. The nuanced phenotypes of VSMCs, modulated by gene regulation and environmental cues, remain pivotal. Essential markers like alpha-SMA, myosin heavy chain, and calponin regulate VSMC migration and contraction, exhibiting diminished expression during VSMC de-differentiation and proliferation. p27^kip^, a CDK inhibitor, shows promise in regulating VSMC proliferation and appears associated with TNF-α-induced pathways impacting unstable plaques. Oncostatin M (OSM), an IL-6 family cytokine, correlates with MMP upregulation and foam cell formation, influencing plaque development. Efforts targeting mammalian target of rapamycin (mTOR) inhibition, notably using rapamycin and its analogs, demonstrate potential but pose challenges due to associated adverse effects. Exploration of the impact of p27^kip^ impact on plaque macrophages presents promising avenues, yet its complete therapeutic potential remains untapped. Similarly, while OSM has exhibited potential in inducing cell cycle arrest via p27^kip^, direct links necessitate further investigation. This critical review discusses the role of mTOR, p27^kip^, and OSM in VSMC proliferation and differentiation followed by the therapeutic potential of targeting these mediators in atherosclerosis to attenuate plaque vulnerability.

## Introduction:

Atherosclerosis, the hardening of the arteries, is a life-threatening contributor to coronary artery diseases (CAD) that affects more than 3 million people in the U.S per year. During atherosclerosis, fatty deposits of cholesterol, fibrin, fats, and waste products build up inside the arterial wallet at the site of damaged inner lining of the artery [1]. Specifically, the cholesterol builds up in the damaged endothelium, called fatty streak, where the cholesterol can become oxidized. The oxidation of cholesterol in this build-up results in an inflammatory response where the monocytes travel to the site and are activated/differentiated into macrophages. The macrophages digest the cholesterol molecules, specifically low-density lipoproteins (LDL), and transform into foam cells contributing to plaque formation and progression. As plaques increase in size, the arterial wall thickens and hardens and there will be increased accumulation of oxidized LDL (ox-LDL). Deposition of ox-LDL, infiltration of immune cells, and secretion of inflammatory cytokines from these immune cells results in inflammation in the plaque. Increased inflammation in the plaque resulting in activation of various inflammatory mediators including toll-like receptors, triggering receptor expressed on myeloid cells, and receptor for advanced glycation end-products and secretion of high mobility group box protein (HMGB)-1 and S100 proteins contribute to plaque vulnerability. These vulnerable plaques can rupture, forming a blood clot in the artery to reduce the blood and oxygen supply to the vital organs [1-8]. During plaque development, the vascular smooth muscle cells (VSMC) within the arterial wall migrate towards the intimal layer to the surface of the plaque and contribute to the formation of a fibrous cap covering the plaque. These caps can rupture and expose the plaques to the blood stream resulting in the formation of blood clot (thrombus) decreasing the blood flow. The complex interaction of these processes, excessive fibrosis of the intima, fatty plaque formation, proliferation of VSMCs and migration of WBCs in response to inflammation characterizes vulnerable plaques in atherosclerosis [9-11].

Plaques can be vulnerable and stable depending on its core composition and thickness of the fibrous cap. Vulnerable plaques are defined by having a rich-lipid core, increased plaque inflammation, positive vascular remodeling, and a thin fibrous cap which can rupture easily causing the activation of platelets [12]. Accumulation of leukocytes and fats in the intima promotes vulnerable plaques. Upon rupture, vulnerable plaques promote blood clotting (thrombosis) aggressively. Stable plaques, unlike vulnerable plaques, have a thick fibrous cap and a small lipid core; thus, less likely to rupture. Ruptured plaques can lead to the blockage of the coronary artery, causing myocardial infarction (MI). In transient ischemic attack (TIA), there is a temporary disruption in the blood flow to the brain due to plaques, resulting in symptoms associated with strokes that last less than an hour. Thus, promoting the stability of the plaques will attenuate cardiovascular events such as MI and TIA [13-15].

Vascular smooth muscle cells (VSMCs) play major roles in early and late-stage atherosclerosis. VSMCs proliferation has been associated with stabilization of plaques in early phase of atherosclerosis while decreased number of VSMCs contribute to thin fibrous cap and plaque vulnerability. Recent studies in VSMC transcriptomics have demonstrated different VSMC phenotypes that play various roles in plaque formation. These roles may be due to a phenotypic switch due to a loss of genes for contractile proteins, such as alpha smooth muscle actin 2, and a gain of genes for secretion, migration, and proliferation. Phenotype switching appears to be regulated by multiple factors such as transcriptional and post-transcriptional regulation, epigenetic modifications, and environmental stimuli [10, 16-18]. Thus, the overall content of VSMCs determines the stability of atherosclerotic plaques through migration, proliferation, and secretion and targeting VSMCs phenotypic switch and proliferation may have therapeutic potential. Although VSMC migration from the tunica media to tunica intima has been a well-established component of atherosclerosis pathogenesis, the exact mechanisms and order in relation to VSMC proliferation have not been fully elucidated [19]. Previous studies have demonstrated that VSMC proliferation is associated with blood vessel injury which damages the internal elastic lamina. However, further studies demonstrated low proliferation indices for VSMCs extracted from late-stage atherosclerotic plaques. This is further explained by phenotype tracing, demonstrating VSMC proliferation begins in the tunica media. The progeny cells then migrate to the intima and further divide into oligoclonal progeny [20].

**Figure 1:**
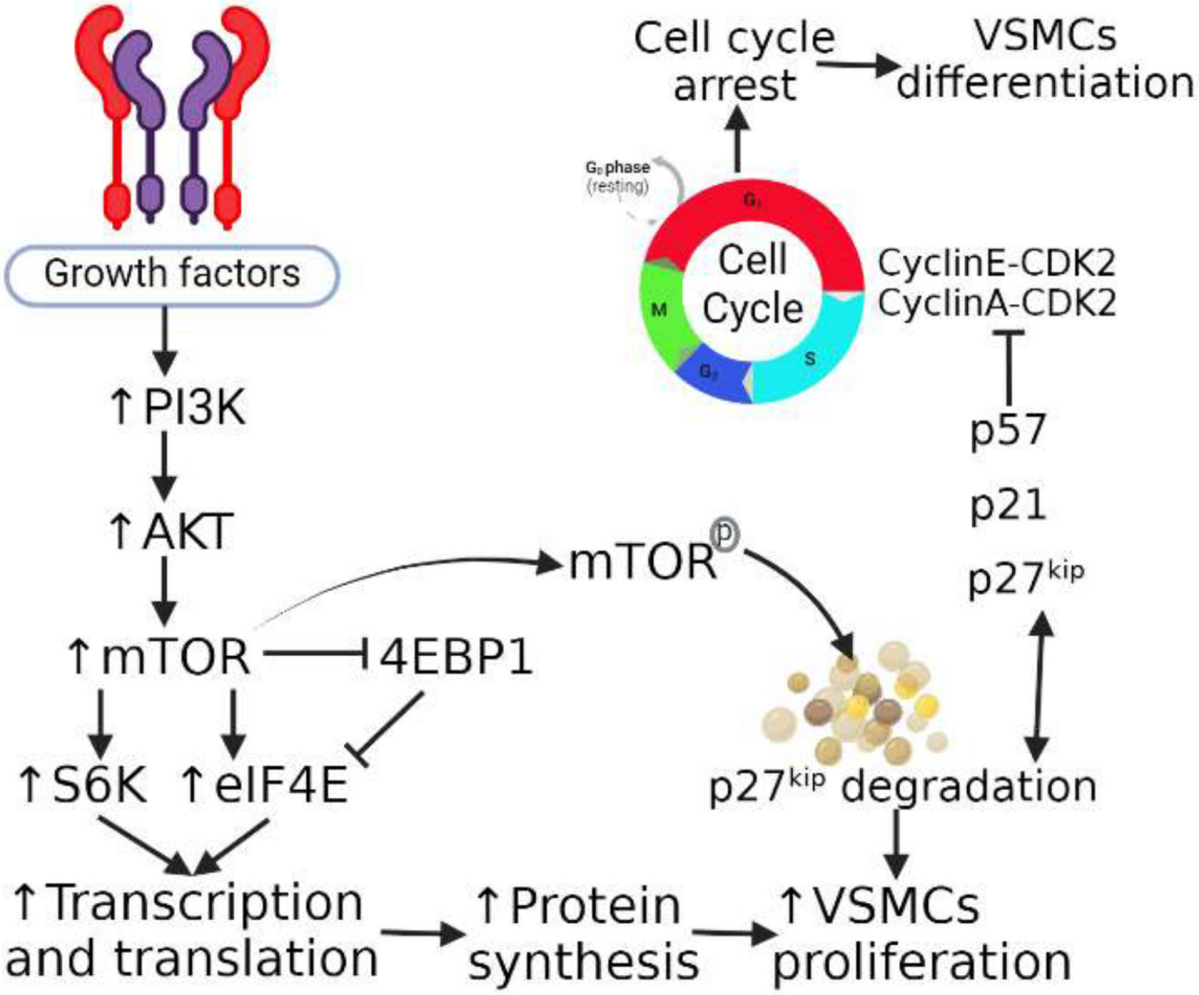
The mammalian target of rapamycin (mTOR), p27kip, and VSMC proliferation and differentiation regulation. mTOR activation via phosphoinositide 3-kinases (PI3K)- protein kinase B (AKT) pathway results in increased protein synthesis resulting in vascular smooth muscle cells (VSMCs) proliferation. mTOR activation also results in p27kip degradation which also causes VSMCs proliferation. An undegraded p27kip inhibits cyclins resulting in cell cycle arrest and this causes VSMCs differentiation.

VSMC migration and proliferation operate in coordination via regulatory protein markers. These include alpha smooth muscle actin (α-SMA), myosin heavy chain, and calponin. These markers are necessary for migration, contraction, and structural integrity, expression of these markers decrease as VSMCs de-differentiate and proliferate [21]. De-differentiation occurs in response to a multitude of factors, including endothelial damage, growth factor exposure, and contact changes with the extracellular matrix. Differentiation of VSMCs requires inhibition of the cell cycle via inhibitors of cyclin-dependent kinase (CDK) and cyclin, which include p21 and p27^kip^. Furthermore, VSMCs that undergo differentiation still retain the ability to de-differentiate in response to injury [21] (Figure 1). This suggests that understanding VSMCs proliferation and differentiation and its regulating involving p27kip will enhance our understanding to develop novel therapeutic strategies [22-24].

## Methods

A literature search was conducted using PubMed and Google Scholar. Articles were selected for inclusion based on keywords, alone or in combination, such as vascular smooth muscle cells, proliferation, differentiation, cell cycle, cyclins, mTOR, p27^kip^, and oncostatin M. The article selection in this review article was based on the article title and abstract, following which the full-text articles were reviewed and included in the bibliography. The search was limited to peer-reviewed articles in the English language only. The duplicate articles, only abstracts, and non-English articles were excluded during the literature search following PRISMA guidelines.

### mTOR, p27^kip^, and OSM and VSMC regulation

The mammalian target of rapamycin (mTOR) is involved in VSMCs proliferation via ribosomal protein S6 kinase beta-1 (p70S6K) signaling pathway and inhibition of angiotensin II receptor type 1 (AT1-R) involving AMP-activated protein kinase (AMPK) [25]. Activation of E2F and mTOR synergistically promote proliferation of VSMCs [26]. The macrolide antibiotic rapamycin has been previously shown to inhibit VSMC proliferation and migration by inducing differentiation of primary VSMCs. Rapamycin acts by binding to FKBPP12, forming a protein complex that inhibits the mammalian target of rapamycin (mTOR). mTOR is a protein kinase involved in signaling pathways that regulate protein synthesis. In one study it was demonstrated that inhibition of mTOR with rapamycin in bovine thoracic aortic VSMCs increased expression of markers associated with the VSMC differentiation, including alpha-SMA and calponin [21]. Moreover, this treatment also induced expression of CDK inhibitors p27^kip^ and p21^cip^ with similar kinetics to those of the expression of the differentiation markers. Therefore it is believed that differentiation and proliferation are regulated in a coordinated manner [14].

p27^kip^, a cyclin-dependent kinase inhibitor, has been shown in prior studies to play a regulatory role in vascular smooth muscle proliferation. To progress through the cell cycle and proliferate, cyclins must interact with CDKs to shift the cell from the G1 to S phase, leading to mitosis [27]. CDK-cyclin complexes drive transcription of proteins necessary for the cell cycle to continue while CDK inhibitors cause arrest in G1 phase and limit proliferation. In a study of mouse model pulmonary artery vascular smooth muscle, p27^kip^ decreased in expression in response to serum stimulation, eventually leading to increased mitogenesis. In contrast, models where p27^kip^ was restored showed decreased phosphorylation of Rb, a cyclin E and CDK2 substrate, with decreased mitogenesis [28,29]. Another study utilizing mouse model neural progenitor cells showed that cell proliferation was more abundant in p27^kip^ knockout mice compared to wild type controls in both homeostatic and ischemic conditions [30]. Further, deficiency of p27 is associated with enhanced inflammatory and proliferative response in hematopoietic progenitor cells [31] and increase neointimal macrophages and atherosclerosis progression. This suggests that CDK inhibitor p27^kip^ controls cell proliferation and attenuating p27^kip^ in developing plaque may attenuate plaque progression by decreasing VSMCs proliferation. This notion is supported by the fact that mitochondrial p27 restores the impaired α-SMA upregulation, a marker of VSMC proliferation, in a p27 deficient mice [32] suggesting the probable role of inhibiting p27 to attenuate VSMCs proliferation. However, it should be noted that a rapid reduction in in p27-phospho-Ser10 levels in the early phase of plaque development (atherosclerosis) results in an early plaque build-up involving RhoA/ROCK-induced integrin expression in the endothelial cells accompanied with enhanced leukocyte recruitment [33]. This suggests that the beneficial effect of inhibiting p27 is phase dependent.

p27^kip^ has been implicated in the Forkhead box subclass O (FoxO) transcription factor pathway in response to the inflammatory modulator tumor necrosis factor alpha (TNF-α) [34]. TNF-α can induce proliferation or apoptosis in human VSMCs depending on phenotype [35]. Previous work had shown that FoxO1 overexpression leads to increased transcription of p27^kip^. Additionally, past studies implicated FoxO1 in differentiation and cell cycle of neointimal hyperplasia in rat model carotid arteries injured via balloon. It was found that in cultured carotid endarterectomy human specimens, TNF-α induced the expression of FoxO1 nuclear protein and therefore p27^kip^, leading to cell cycle arrest in asymptomatic plaques. This arrest may also be coordinated with a FoxO1-induced increase in Caspase-3 expression, leading to apoptosis and therefore inhibition of neointimal hyperplasia [36]. Therefore, the TNF-α induced pathway involving FoxO1 expression may be a mechanism to better study unstable atherosclerotic plaques.

Oncostatin M (OSM) is an IL-6 family cytokine which modulates pro-inflammatory effects through JAK/STAT signaling pathways. OSM results in downstream upregulation of mixed metalloproteinases (MMPs) such as MMP-9 through Mitogen-Activated Protein Kinase Kinase-Extracellular Signal-Regulated Kinase Pathway (MAPK-ERK). MMPs are effectors of the inflammatory response to intimal injury which can lead to plaque formation. Additionally, the MAPK-ERK pathways promotes formation of foam cells, which further secrete MMPs [37,38]. Further, OSM produced by immune cells has a context dependent effect on cellular processes such as differentiation, hematopoiesis, cell proliferation and cell survival. OSM signaling is initiated by Binding of OSM with its receptors type I (LIFRβ/gp130) or type II (OSMRβ/gp130) complexes activates OSM leading to the increased expression of JAK/STAT, MAPK and PI3K. In addition to triggering plaque formation, OSM has also been shown to upregulate p27^kip^ mRNA. A previous study has shown that through signal transduction through the gp130 receptor, OSM induce increased expression of p27^kip^ in melanoma cells [39]. Moreover, this was further observed in HepG2 hepatoma cells in which signaling through the OSM receptor (OSMR) resulted in accumulation of p27^kip^ and cell cycle arrest [40]. While these results establish a relationship between OSM and p27^kip^, there has yet to be a conclusive investigation into this link in the context of plaque stability.

### Therapeutic Targets

While we have discussed multiple markers and pathways involved in atherogenesis, therapeutic targets are still being explored. The mammalian target of rapamycin (mTOR) is a constitutively active serine threonine kinase that is a key part of two separate multiprotein complexes: mTORC1 and mTORC2 [41]. mTORC1 under physiologic conditions promotes lipogenesis through signaling with cyclin-dependent kinases, S6 kinase 1, and various other signaling pathways. Inhibition of mTORC1 was shown to promote longevity in mice models while overexpression was associated with cancers [42,43]. mTORC2 is less studied but has been shown to promote insulin resistance when inhibited [44]. Recent studies have demonstrated coordination and crosstalk between mTORC1 and mTORC2.

In addition to direct inhibition of mTOR using rapamycin, other therapies have been developed to target its involvement in the atherosclerotic pathogenesis. Rapamycin has been used in drug eluting stents (DES) and cancer treatment for its anti-proliferative effects. While it has a strong inhibitory effect on mTORC1 and less potent effect on mTORC2 applicable only when used chronically, rapamycin has poor bioavailability and long half-life. Thus, rapamycin analogs (rapalogs) such as everolimus and zotarolimus have been developed with improved pharmacodynamic and pharmacokinetic properties. Further studies with everolimus in DES in cholesterol-fueled atherosclerotic rabbit arteries demonstrated reduction of plaque macrophages, possibly due to activation of autophagy noted by the presence of massive vacuoles [45]. To better account for the systemic nature of atherosclerosis and its inflammatory pathways, everolimus and other rapalogs have been administered orally in humans after bare metal stents with 3 years follow up showing minimal side effects and good tolerance [41]. Despite these results, rapalog usage can lead to adverse effects due to suppression of mTORC1. Inhibition of the proliferative effects of mTORC1 in rapalog-coated DES therapy can prevent reendothelialization of the targeted vessel, increasing the risk of stent thrombosis [46]. Additionally, inhibition of mTORC1 also leads to inhibition of lipases and LDL receptors, leading to hypercholesterolemia in mice models. Furthermore, suppression of mTORC2 as noted before can lead to insulin resistance and subsequent hyperglycemia. Therefore, combination drug therapy with statins and metformin has been proposed to mitigate the adverse effects of dyslipidemia and glucose intolerance respectively [47]. Other strategies suggest an intermittent administration of rapalogs to prevent mTORC1 resistance.

While p27^kip^ has shown potential as a therapeutic target for plaque formation, this area of research has yet to be fully explored. Aside from its role in cell cycle regulation, p27^kip1^ has been used in a study focused on inhibition of plaque macrophages in the pathogenesis of atherosclerosis utilizing the cyclin dependent kinase inhibitor p27^kip^. Macrophages can be activated by various factors, including oxidized LDL, leading to secretion of pro-inflammatory cytokines and growth factors. In advance-stage lesions, foam cells can form by dysregulation of lipid metabolism by macrophages. Proliferation and overall count of foam cells is positively correlated with plaque formation [48]. Thus, inhibition of plaque macrophages has been an area of focus in the inhibition of atherosclerosis. In the study of interest, transgenic mice expressing human p27^kip^ in macrophages were generated and crossed with mice lacking ApoE [48]. With an abundance of the CDK inhibitor, resulting macrophage cell cycles could be arrested despite exposure to intimal injury. The resulting progeny showed decreased plaque formation and inflammatory response with increased stability of existing plaques. The researchers noted smaller necrotic cores, thicker fibrous caps, and increased extracellular matrices in these plaques. Thus, future research may examine further uses of p27^kip^ expression in halting inflammatory processes by directly impacting the proliferation of inflammatory effector cells. Targeting p27 in atherosclerosis is also supported by the findings that p27Kip1 protects against diet-induced atherosclerosis [49] and supported by the fact that early deletion of p27 promote atherosclerosis [33]. Together suggesting that p27 is necessary to attenuate atherosclerosis progression in the early phase while detrimental in later phases.

## Conclusion

Atherosclerosis is a chronic inflammatory disorder regulated by multiple signaling pathways modulating plaque formation and stability. Regulation of vascular smooth muscle proliferation and migration plays an important role as the smooth muscle phenotypes impact the stability and longevity of the plaque. mTOR is a ubiquitous protein kinase involved in pathways leading to cell growth and RNA transcription. Its inhibition by the macrolide rapamycin also impacts regulators of cell proliferation such as the cyclin-dependent kinase inhibitor p27^kip^. Attempts to contextualize these two molecules in the setting of atherosclerosis have also implicated the IL-6 class cytokine oncostatin M. However, given the multifactorial nature of atherosclerosis, a direct link between these three effectors is still being investigated. While therapeutics individually targeting or utilizing these molecules show promise, future studies may benefit from studying the pathways and crosstalk that intertwine them.
